# Evaluation of the possible impact of the fear of hypoglycemia on diabetes management in children and adolescents with type 1 diabetes mellitus and their parents: a cross-sectional study

**DOI:** 10.1007/s42000-024-00560-z

**Published:** 2024-04-12

**Authors:** Ourania Andreopoulou, Eirini Kostopoulou, Eleni Kotanidou, Sophia Daskalaki, Angeliki Vakka, Assimina Galli-Tsinopoulou, Bessie E Spiliotis

**Affiliations:** 1 Department of Psychiatry, University of Patras Medical School, Rio, 26500 Greece; 2 Division of Pediatric Endocrinology and Diabetes, Department of Pediatrics, University of Patras School of Medicine, Patras, 26500 Greece; 3 Unit of Pediatric and Adolescent Diabetes Mellitus, Second Department of Pediatrics, School of Medicine, Faculty of Health Sciences, Aristotle University of Thessaloniki, AHEPA University Hospital, Thessaloniki, Greece; 4 Department of Electrical and Computer Engineering, School of Engineering, University of Patras, Patras, 26500 Greece

**Keywords:** Fear of hypoglycemia, Type 1 diabetes mellitus, Hypoglycemia fear survey, Reliability

## Abstract

**Purpose:**

Hypoglycemia represents a significant source of anxiety for children with type 1 diabetes mellitus (T1DM) and their caretakers. Fear of hypoglycemia (FoH) was measured in children and adolescents with T1DM as well as in their parents using an established research instrument, the Hypoglycemia Fear Survey (HFS).

**Methods:**

This is a two-center, cross-sectional study involving 100 children and adolescents aged 6–18 years old diagnosed with T1DM. One parent of each child also participated in the study. The participants, who were recruited from two different pediatric endocrine outpatient clinics, were asked to complete the translated Greek version of the HFS, which includes one version for children (C-HFS) and one for parents (P-HFS). The association of the questionnaire responses with subjects’ characteristics, such as current age, age at diagnosis, duration of diabetes, HbA1c levels, and mode of diabetes treatment were assessed.

**Results:**

Parents exhibited significantly higher mean HFS scores than their children. No significant correlation was found between the P-HFS or the C-HFS scores and the age of the children, duration of diabetes, HbA1c, or mode of treatment.

**Conclusion:**

The finding that parents experience higher levels of FoH compared to their children emphasizes the importance of healthcare providers to screen parental FoH and focus on approaches to support them in order to reduce their psychological burden, thus optimizing children’s diabetes management.

**Supplementary Information:**

The online version contains supplementary material available at 10.1007/s42000-024-00560-z.

## Introduction

Hypoglycemia constitutes the commonest acute complication of type 1 diabetes mellitus (T1DM) [1]. Aggressive HbA1c goals may increase the frequency of hypoglycemia, which represents a major limiting factor in the glycemic control of T1DM [2]. Due to its high frequency and unpredictability, hypoglycemia is a significant source of anxiety for both patients and their caretakers.

Fear of hypoglycemia (FoH) is a specific fear caused by the risk and/or occurrence of hypoglycemia [3] and is associated with the frequency of severe hypoglycemic episodes [4], particularly during the past 3 months [3]. While to some degree this fear is adaptive, it may disrupt daily activities, such as sleep and exercise. It can also impair optimal diabetes management and quality of life through obsessive self-monitoring, dependence on others, frustration, and behavior that tend to keep blood glucose levels high [5–8]. FoH has been recorded in family members of both pediatric and adult patients with T1DM, and, as may be expected, distressing past experiences of hypoglycemia that involved either loss of consciousness or any hypoglycemic-related traumatic event are often associated with even higher levels of fear [6, 9, 10]. Furthermore, research suggests that older age, female gender, and longer duration of T1DM can result in children and their families exhibiting higher levels of FoH [11]. According to a study by Patton et al., the commonest reported parental fear, i.e., concerning hypoglycemia, involved episodes during the night, while the next most common fear involved episodes when the child was not supervised by their parents [12].

FoH in children and their parents can be evaluated using the Hypoglycemia Fear Survey-II (HFS-II), a widely used research instrument translated into many languages that has demonstrated reliability and validity [13]. Our team has recently translated the HFS into the Greek language and has confirmed its usefulness as a reliable and valid research tool for assessing the fear of hypoglycemia in Greek children and adolescents with T1DM and their parents [14].

The aim of the current study was to evaluate the association between self-reported FoH in children and adolescents with T1DM or their parents and demographic or disease-related factors, including the child’s age, duration of T1DM, recent HbA1c, mode of treatment, and frequency of hypoglycemic episodes. This is the first study, to our knowledge, carried out in Greece addressing this crucial component of the possible impact of FoH on diabetes management.

## Materials and methods

### Demographics

This is a cross-sectional study conducted between January 2019 and July 2020 in a sample of 100 Greek children and adolescents, aged 6 to 18 years old, diagnosed with type 1 diabetes mellitus (T1DM) at least 3 months before the initiation of the study. Fifty-four of the participants were male. Participants were recruited from two different centers where they were being actively followed up, namely: (1) the Outpatient Clinics of the Division of Pediatric Endocrinology and Diabetes of the University General Hospital of Patras in Southern Greece and (2) the Outpatient Clinics of the Unit of Pediatric and Adolescent Diabetes, 2nd Department of Pediatrics, AHEPA General Hospital of Thessaloniki in Northern Greece. The duration of diabetes varied from 3 months to 16.73 years (mean ± sd: 5.05 ± 4.09). One parent involved in each participant’s diabetes care also participated. All participants could read and write in Greek and had the mental ability to complete the questionnaires. Exclusionary criteria included significant comorbidity in adolescents that could affect psychosocial status, quality of life, or FoH (e.g., cystic fibrosis), and cognitive or learning disabilities of the child or the parent (e.g., inability to read) that would preclude their ability to complete the study protocol.

### Questionnaires

All participants were asked to complete the translated Greek language version of the *Hypoglycemia Fear Survey* (HFS), which is the most well-established instrument assessing FoH [15].

### Scale translation

Permission for this study was obtained from Dr. Linda Gonder-Frederick, the original co-author and co-creator of the scale, through personal communication via email. Two forward translations were produced from the original language (source language) to the target language. Bilingual translators (EK & OA), whose mother tongue is the target language, produced the two independent translations. The two translators had different academic profiles and each produced a written report. The translations were then compared and discrepancies between them were resolved through interactive discussions between the translators. This last step converged to a single translation. Finally, a third translator, BES, who is an expert in the field, a native English speaker, and blind to the original English version, back-translated the instrument to its original language to check validity by ensuring that the translation reflected the same item content as the original version. Source and back-translated questionnaires were checked for all such equivalences. Consensus was reached on the items and the final translation was reviewed and approved by Dr L. Gonder-Frederick.

Two versions of the HFS were used, one for children with T1DM (C-HFS) and one for their parents (P-HFS). The parent version (P-HFS) is a 26-item questionnaire used to measure parents’ FoH [16]. It is composed of two subscales, the Behavior subscale and the Worry subscale. The Behavior subscale includes 11 items describing diabetes self-management behaviors aimed at avoiding hypoglycemia and its negative consequences (e.g., keeping blood glucose (BG) higher when the adolescent is alone). The Worry subscale includes 15 items describing different anxiety-provoking aspects of hypoglycemia (e.g., P-HFS items are rated on a 5-point Likert scale, ranging from 0 (never) to 4 (almost always). A total HFS score is obtained by adding the Behavior and Worry subscale items. The P-HFS also includes the additional Hypoglycemia History Questionnaire (Part–II) regarding the frequency of hypoglycemic episodes of the child over the past 1, 6, or 12 months while the parent was absent. The child version of HFS (C-HFS) is a 25-item self-report measure of children’s FoH. The C-HFS is similar to the P-HFS and comprises a 10-item Behavior subscale and a 15-item Worry subscale that are scored on the same 5-point Likert scale as the P-HFS [16].

### Procedure

In addition to their responses to the HFS questionnaire, demographic and disease-specific data, such as the current age of the participants, the most recent HbA1c, and the duration of diabetes, were also recorded for all participants in the study. The participants were recruited by the physicians of the Pediatric Endocrine Division during their visit to the hospital. HbA1c was measured on the same day as the completion of the HFS questionnaire for the majority of the children and within 3 weeks from the completion of the questionnaire for a small minority. The mode of treatment, i.e., Multiple Dose Insulin Injection Therapy (MDI) or insulin administration via an insulin pump, was also recorded. Parents and children were additionally asked to fill in a medical history form and report the number of severe, medium, or mild hypoglycemic episodes that children experienced during the previous year, past semester, or past month, respectively. Mild hypoglycemia (MiH) was defined as BG < 70 mg/dl which did not impair the ability to function. Medium hypoglycemia (MeH) was defined as BG so low that it interfered with the child’s ability to function, but the child did not become so mentally disoriented that self-treatment was not possible. Severe hypoglycemia (SH) was defined as BG resulting in neuroglycopenia that interfered with the child’s ability to self-treat due to mental disorientation, unconsciousness, or seizures. Lastly, the levels of FoH between younger (6–12 years old) and older (> 12 years old) children and between children with optimum glycemic control (HbA1c < 7.0%) and those with poor glycemic control (HbA1c > 7.0%) were compared.

The participants were also asked to complete the Pediatric Quality of Life Inventory (PedsQLTM 3.0 Diabetes Module), a widely used research tool that measures the health-related quality of life (HRQOL) of families with children aged 2–18 years old diagnosed with T1DM [17]. The correlation coefficients between the different subscales of the PedsQL™ 3.0 Diabetes Module and the scores of the HFS scales/subscales were estimated [14].

The study was approved by the Research Ethics Committee of the University Hospital of Patras (IRB number: 348/9.5.2017) and is in full accordance with the 1975 Declaration of Helsinki, as revised in 2013. Written informed consent was obtained from the parents and informed assent from the participating children and adolescents.

## Results

The characteristics of all participants, including current age, age at onset of T1DM, duration of T1DM, and recent measurement of HbA1c, are summarized in Table 1. The sample was balanced between genders.

**Table 1:** Sample characteristics of the studied children and adolescents (*N* = 100): current age, age at onset of T1DM, duration of T1DM, recent HbA1c

Characteristic		Min	Max	Mean	Std Dev.
***Current age (years)***		5.8	20.0	13.70	3.35
	Male	6.3	20.0	13.71	3.53
	Female	5.8	18.4	13.70	3.18
***Age at onset of T1DM (years) *** ^*^		1.25	16.50	8.65	3.91
	Male	1.25	16.50	8.71	3.73
	Female	1.25	15.90	8.59	4.16
***Duration of T1DM (years)***		0.25	16.73	5.05	4.09
	Male	0.25	15.00	5.00	4.01
	Female	0.25	16.73	5.11	4.23
***Recent HbA1c (%)*** ^**^		5.0	9.4	7.27	0.98
	Male	5.0	9.0	7.08	0.98
	Female	5.4	9.4	7.49	0.95

^*^ T1DM: Type 1 diabetes mellitus, ^**^ HbA1c: Glycosylated hemoglobin.

**Fig. 1:**
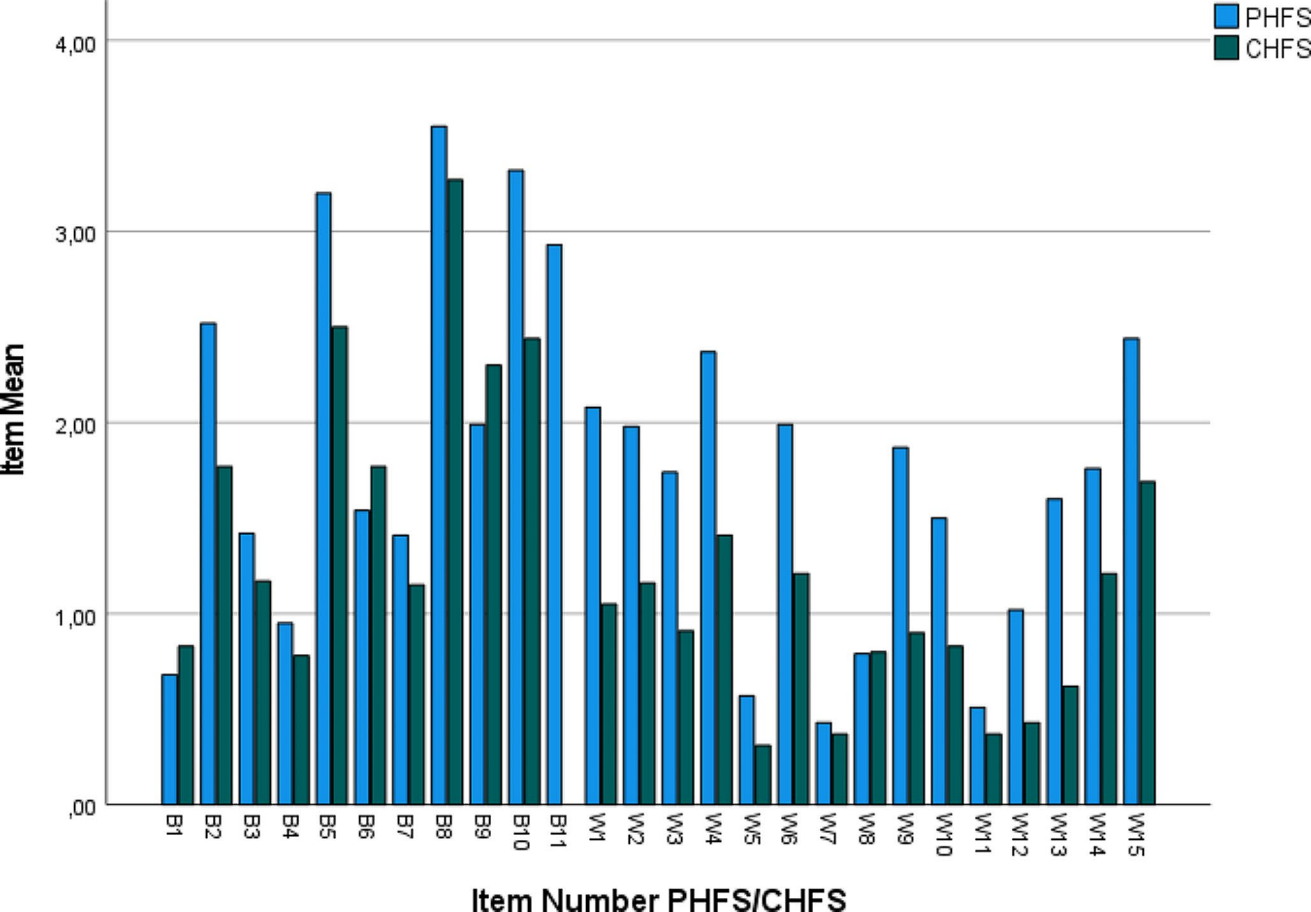
Mean scores of different items of the P-HFS and C-HFS scales

Based on their mean scores in the HFS, the parents scored higher than the children in most items (Fig. 1). B1 (I eat large bedtime snacks), B6 (I decrease my insulin dose whenever I think that my blood sugar is very low), and B9 (I restrict my activity whenever I think that I may have low blood sugar) were the only items where the children’s mean score was higher than the corresponding mean score of their parents. In totality, the mean scores for parents were significantly higher for both subscales and the Total scale (paired T-test, *p* < 0.0005) compared to the children’s corresponding scores (Table 2).

**Table 2:** Mean scores and standard deviations for HFS (Behavior, Worry and Total scales) for the studied parents and children

	P-HFS		C-HFS	
	Mean	Std Dev.	Mean	Std Dev.
*HFS Behavior*	2.14	0.56	1.80	0.74
*HFS Worry*	1.52	1.04	0.88	0.65
*HFS Total*	1.78	0.69	1.25	0.53

The analysis of the current study was performed using the mean scores for all subscales or Total scales instead of the total scores. This facilitated the handling of all missing values. Eighty-four out of 100 parents responded to all items, 12 missed one item, three missed two or three items, and only one missed six items from the Behavior subscale. For the children, the missing values were fewer, i.e., 89 out of 100 responded to all 25 items, ten missed one item either from the Behavior or Worry subscale and only one missed two items from the Worry subscale.

### Correlations between P-HFS/C-HFS scores and patient characteristics

#### HFS scores and children’s current age

No significant correlation was observed between the P-HFS scores, both the two subscales and the Total scale, and the current age of the child (*r* = 0.023, *p* = 0.820; *r*=-0.075, *p* = 0.455; *r*=-0.057, *p* = 0.575; for Behavior, Worry, and Total scale, respectively). Similarly, there was no significant correlation between the C-HFS scores (both the two subscales and the Total scale) and the age of the child (*r*=-0.065, *p* = 0.521; *r*=-0.076, *p* = 0.451; *r*=-0.093, *p* = 0.358; for Behavior, Worry, and Total scale, respectively).

#### HFS scores and duration of diabetes

No significant correlation was found between the P-HFS scores, both the two subscales and the Total scale, and duration of diabetes (*r* = 0.01, *p* = 0.920; *r*=-0.112, *p* = 0.266; *r*=-0.089, *p* = 0.378; for Behavior, Worry, and Total scale, respectively). Similarly, there was no significant correlation between the C-HFS scores (both the two subscales and the Total scale) and the duration of diabetes (*r*=-0.153, *p* = 0.130; *r*=-0.034, *p* = 0.740; *r*=-0.110, *p* = 0.276; for Behavior, Worry, and Total scale, respectively).

#### HFS scores and recent HbA1c measurement

There was no significant correlation between the P-HFS scores (both the two subscales and the Total scale) and children’s HbA1c levels at the time of completion of the questionnaire (*r* = 0.149, *p* = 0.140; *r* = 0.145, *p* = 0.150; *r* = 0.181, *p* = 0.071; for Behavior, Worry, and Total scale, respectively). There was also no significant correlation between the C-HFS scores (both the two subscales and the Total scale) and children’s HbA1c levels at the time of completion of the questionnaire (*r*=-0.177, *p* = 0.079; *r* = 0.041, *p* = 0.687; *r*=-0.070, *p* = 0.488; for Behavior, Worry, and Total scale, respectively).

### Comparisons of the HFS scores in different subgroups

#### HFS scores in younger versus older children (current age)

The parents of younger children scored on average slightly higher than the parents of adolescents; however, this difference was not significant for any of the subscales or the Total scale (Table 3). According to the independent samples T-test, there was no significant difference between the two age groups [t(98) = 0.457, *p* = 0.649; t(98) = 0.711, *p* = 0.479; t(98) = 0.758, *p* = 0.450].

**Table 4:** Mean scores and standard deviations (in parentheses) of Behavior, Worry, and Total HFS scales for parents and children according to the frequency of severe, moderate, and mild hypoglycemic episodes. SH: severe hypoglycemic episode, MeH: moderate hypoglycemic episode, MiH: mild hypoglycemic episode

P-HFS	*SH ≤ 1 (* *N* * = 83)*	*SH > 1 *(*N = 12)*
*Mean HFS Behavior*	2.12 (0.54)	2.10 (0.54)
*Mean HFS Worry*	1.54 (1.01)	1.30 (1.14)
*Mean Total HFS*	1.79 (0.67)	1.64 (0.80)
*C-HFS*	**SH ≤ 1 (** *N* ** = 76)**	**SH > 1 (** *N* ** = 15)**
*Mean HFS Behavior*	1.89* (0.70)	1.17* (0.70)
*Mean HFS Worry*	0.92 (0.68)	0.77 (0.62)
*Mean Total HFS*	1.31* (0.51)	0.94* (0.60)
*P-HFS*	**MeH ≤ 2 (** *N* ** = 60)**	**MeH > 2 (** *N* ** = 34)**
*Mean HFS Behavior*	2.16 (0.59)	2.02 (0.45)
*Mean HFS Worry*	1.47 (0.93)	1.55 (1.19)
*Mean Total HFS*	1.76 (0.63)	1.76 (0.78)
*C-HFS*	**MeH ≤ 2 (** *N* ** = 56)**	**MeH > 2 (** *N* ** = 35)**
*Mean HFS Behavior*	1.97** (0.73)	1.47** (0.67)
*Mean HFS Worry*	0.96 (0.72)	0.79 (0.58)
*Mean Total HFS*	1.36** (0.55)	1.07** (0.48)
*P-HFS*	**MiH ≤ 3 (** *N* ** = 48)**	**MiH > 3 (** *N* ** = 48)**
*Mean HFS Behavior*	2.04 (0.58)	2.22 (0.49)
*Mean HFS Worry*	1.36 (1.02)	1.74 (1.04)
*Mean Total HFS*	1.65 (0.70)	1.95 (0.68)
*C-HFS*	**MiH ≤ 3 (** *N* ** = 43)**	**MiH > 3 (** *N* ** = 49)**
*Mean HFS Behavior*	1.72 (0.79)	1.81 (0.71)
*Mean HFS Worry*	0.93 (0.77)	0.86 (0.57)
*Mean Total HFS*	1.25 (0.56)	1.24 (0.53)

^*Significant difference at the 0.05 level (2−tailed)^

^**Significant difference at the 0.01 level (2−tailed)^.

Children’s scores were significantly lower than those of their parents, although there was no significant difference between the two age groups (Table 3). The results from the corresponding independent samples T-test were as follows: [t(68) = 1.095, *p* = 0.277; t(98)=-0.045, *p* = 0.964; t(98) = 0.506, *p* = 0.614].

**Table 3:** Mean scores and standard deviations (in parentheses) of Behavior, Worry and Total HFS scales for parents and children according to patient’s age group, mode of treatment, good (HbA1c ≤ 7.0%) versus poor (HbA1C > 7.0%) glycaemic control

P-HFS	*Age 6–11 (* *N* * = 28)*	*Age 12–18 (* *N* * = 72)*
***Mean HFS Behavior***	2.19 (0.47)	2.13 (0.59)
*Mean HFS Worry*	1.60 (1.08)	1.48 (1.03)
*Mean Total HFS*	1.85 (0.71)	1.76 (0.69)
*C-HFS*	**Age 6–11 (** *N* * = 28)*	**Age 12–18 (** *N* * = 72)*
*Mean HFS Behavior*	1.85 (0.64)	1.77 (0.78)
*Mean HFS Worry*	0.86 (0.71)	0.89 (0.63)
*Mean Total HFS*	1.26 (0.56)	1.25 (0.52)
*P-HFS*	**MDI (** *N* ** = 85)**	**Pump (** *N* ** = 14)**
*Mean HFS Behavior*	2.12 (0.58)	2.26 (0.47)
*Mean HFS Worry*	1.52 (1.04)	1.57 (1.09)
*Mean Total HFS*	1.78 (0.69)	1.86 (0.76)
*C-HFS*	**MDI (** *N* ** = 85)**	**Pump (** *N* ** = 14)**
*Mean HFS Behavior*	1.80 (0.74)	1.73 (0.72)
*Mean HFS Worry*	0.86 (0.63)	1.02 (0.79)
*Mean Total HFS*	1.24 (0.50)	1.30 (0.68)
*P-HFS*	**HbA1c ≤ 7,0% (** *N* ** = 44)**	**HbA1c > 7,0% (** *N* ** = 56)**
*Mean HFS Behavior*	2.03* (0.61)	2.23* (0.52)
*Mean HFS Worry*	1.37 (1.01)	1.63 (1.05)
*Mean Total HFS*	1.65* (0.69)	1.89* (0.68)
*C-HFS*	**HbA1c ≤ 7.0% (** *N* ** = 44)**	**HbA1c > 7.0% (** *N* ** = 56)**
*Mean HFS Behavior*	2.00** (0.66)	1.63** (0.75)
*Mean HFS Worry*	0.93 (0.72)	0.85 (0.60)
*Mean Total HFS*	1.36* (0.54)	1.16* (0.51)

^*Significant difference at the 0.05 level^

^**Significant difference at the 0.01 level^.

#### HFS scores and mode of treatment (MDI versus insulin pump)

All mean P-HFS scores were slightly, but not significantly, higher for parents of children treated via an insulin pump compared to those of children who are on MDI (Table 3). The results of the independent samples T-test [t(97)=-0.861, *p* = 0.391; t(97)=-0.158, *p* = 0.875; t(97)=-0.406, *p* = 0.686] demonstrated no significant difference between the two groups of parents.

Furthermore, the mean C-HFS scores showed no statistical difference between the two groups. Based on the independent samples T-test, no significant difference was observed for any of the subscales or for the Total scale [t(98) = 0.341, *p* = 0.734; t(98)=-0.824, *p* = 0.412; t(98)=-0.422, *p* = 0.674].

#### HFS scores and HbA1c (HbA1c < 7.0% versus HbA1c ≥ 7.0%)

The average HFS scores for the parents of children with recent measurement of HbA1c > 7.0% were greater than the corresponding average scores of those of children with recent HbA1c measurement ≤ 7.0%, while this was reversed for the children (Table 3). For the parents, the mean Behavior subscale score and the mean Total scale score were both significantly higher (level of significance 0.05) for those with HbA1c > 7.0% according to a right-sided independent samples T-test [t(98)=-1.81, *p* = 0.037; t(98)=-1.75, *p* = 0.042]. For the corresponding mean Worry subscale scores, there was no significant difference between the two groups of parents [t(98)=-1.25, *p* = 0.108]. In contrast, for the children, the mean Behavior subscale score and the mean Total scale score were both significantly higher for those with HbA1c ≤ 7.0% according to a right-sided independent samples T-test t(98) = 2.61, *p* = 0.005; t(98) = 1.88, *p* = 0.032]. Similarly to the parents, for the mean C-HFS Worry subscale scores, there was no significant difference between the two groups [t(98) = 0.57, *p* = 0.284].

#### Effect of hypoglycemia on HFS scores

Supplementary File 1 summarizes the children’s and parents’ responses regarding the frequency of severe, moderate, and mild hypoglycemic episodes during the previous year, previous 6 months, and previous month, respectively.

The mean HFS scores of children and parents grouped according to the number of severe hypoglycemic episodes (≤ 1 versus > 1) over the previous 1 year, moderate episodes (≤ 2 versus > 2) over the previous 6 months, and mild episodes (≤ 3 versus > 3) over the previous 1 month are shown in Table 4.

With regard to severe hypoglycemic episodes and according to the independent samples T-test, there was no significant difference between the mean scores of the parents of the two groups of children [t(93) = 0.81, *p* = 0.935; t(93) = 0.764, *p* = 0.447; t(93) = 0.700, *p* = 0.486; for Behavior, Worry, and Total scale, respectively]. The average scores of children with more than one SH episode during the last year were consistently lower than the corresponding average scores of those with none or one SH episode. According to the independent samples T-test, however, the difference was significant only for the Behavior scale and the Total HFS scale [t(89) = 3.66, *p* < 0.0005; t(89) = 2.50, *p* = 0.014], but not for the Worry subscale [t(89) = 0.766, *p* = 0.446].

Similarly, regarding the moderate hypoglycemic episodes that occurred during the previous 6 months, according to the independent samples T-test, there was no significant difference between the mean HFS scores for the parents of the two groups of children for either subscale or the Total scale [t(84) = 1.278, *p* = 0.205; t(56)=-0.325, *p* = 0.747; t(57) = 0.045, *p* = 0.964]. In contrast, the children who had ≤ 2 MeH exhibited consistently higher average scores compared to those who had > 2 MeH. According to the independent samples T-test, there was a significant difference between the two groups of children for the Behavior subscale and the Total HFS scale [t(89) = 3.22, *p* = 0.002; t(89) = 2.62, *p* = 0.01], but not for the Worry subscale [t(89) = 1.20, *p* = 0.234].

Lastly, with respect to MiH during the previous 1 month, according to the independent samples T-test, there was no significant difference between the scores of any of the HFS subscales or the Total scale for the parents of the two groups of children [t(94)=-1.651, *p* < 0.102; t(94)=-1.816, *p* = 0.073; t(94)=-2.074, *p* = 0.041, respectively], as well as for the children [t(90)=-0.60, *p* < 0.549; t(90) = 0.547, *p* = 0.586; t(90) = 0.069, *p* = 0.945, respectively].

#### HFS scores and PedsQL™ scores

A significant positive correlation was found between the Total HFS scores and the overall PedsQL™ scores (*r* = 0.337, *p* < 0.001; *r* = 0.408, *p* < 0.001 for parents and children, respectively). Furthermore, the average PedsQL™ scores calculated for the upper tertiles of HFS for both parents and children were significantly higher than those in the lower tertiles based on right-sided t-tests [Lower: 0.84 ± 0.42, Upper: 1.20 ± 0.51; *t*(64)=-3.178, *p* = 0.001 (for parents); Lower: 0.65 ± 0.48, Upper: 1.15 ± 0.50; *t*(65)=-4.19, *p* < 0.001 (for children)].

## Discussion

Maintenance of optimal glycemic control and prevention of late microvascular and macrovascular diabetes complications while avoiding episodes of hypoglycemia represents a challenge in the management of T1DM. However, it is not only the uncomfortable and embarrassing nature of the symptoms that makes hypoglycemia a stressful experience, but predominantly the life-threatening nature of the condition. As a result, fear of hypoglycemia brought about by the risk and/or occurrence of low blood glucose levels appears to be a common psychosocial consequence among patients with T1DM [18]. A certain level of fear is beneficial and helps maintain optimum glycemic control, but extreme fear may impair the person’s well-being, self-management, health, and quality of life. Different counteractive strategies may be used by children with T1DM or their parents in order to avoid hypoglycemia due to their fear, including increased intake of carbohydrates, avoidance of physical activity, and administering less insulin than required [19–21]. In this case scenario, the question of potential endangerment of optimal glucose management is raised. Interestingly however, despite a tendency among parents and adolescent patients to seek to keep BG concentrations at higher levels, a clear association between FoH and HbA1c has not been confirmed [16, 22, 23].

In the present study, we attempted to evaluate the FoH in Greek children and adolescents with T1DM and in their parents by using the validated Greek version of the HFS questionnaire. One of the most important findings of the current study was the increased FoH documented in the parents of children with T1DM compared to their children. This is in agreement with findings from other studies [24, 25]. This finding may be of clinical relevance since, according to the literature, fearful parents may intentionally keep their children’s BG high in an effort to prevent hypoglycemia [8]. However, other studies have not found an association between parental FoH and suboptimal glycemic control in pediatric patients [26].

Similarly, in the present study, the FoH among the parents and children was not highly correlated with the HbA1c. Interestingly, nevertheless, parental FoH was significantly higher in the case of children with HbA1c above 7.0%, this involving only the Behavior subscale and the Total scale of HFS. It is reasonable to hypothesize that higher parental fear of hypoglycemia may result in behaviors focused on maintaining higher glucose levels and may account for impaired glucose regulation as expressed by HbA1c levels of above 7.0%. However, causality cannot be established. In addition, a significant difference was documented in the FoH between children with normal HbA1c (≤ 7.0%) and those with elevated HbA1c (> 7.0%). Children with lower HbA1c values (≤ 7.0%) had increased FoH, possibly due to the frequent proximity of their glucose values to hypoglycemia. One could conclude that FoH in both the patient and his/her caretaker is a complex concept and its impact on diabetes management still remains unclear. Several studies support our finding of a lack of correlation between FoH and HbA1c in children with T1DM and/or their parents [12, 27–30], whereas others have shown the opposite [12, 31]. On the other hand, it should be noted that a single HbA1c value is not always indicative of glycemic control as a whole. Moreover, even in the case of consistent HbA1c levels over time, HbA1c alone is not the gold-standard index for the evaluation of glycemic control as it represents the mean glucose concentrations of the past few months and provides no information about glucose variability. Even if parents with high FoH proactively tend to maintain glucose levels at a higher level, the equilibrium between high glucose levels and the total number of episodes of hypoglycemia, which is associated with many other factors, such as the insulin dose, the rate and intensity of exercise, and correct carbohydrate counting, may affect HbA1c levels.

Another interesting finding of the present study was the absence of correlation between FoH and patients’ age. Even though parents of younger children scored on average a little higher than parents of adolescents, the difference was not significant. Similarly, no significant difference was observed in the FoH reported between the younger and the older children. It could be hypothesized that having a younger child with T1DM may exert more psychological pressure on the parents due to the child’s lower capacity to manage difficult situations related to diabetes, such as a severe hypoglycemic episode. This hypothesis is supported by a study by Gonder-Frederick et al. which demonstrated that behavior scores were higher among the parents of younger children, aged 6–11 years old, compared to the parents of older children, aged 12–18 years old [27]. In the same study, the Worry scores of older children, aged 9–18 years old, were higher than those of younger children (6–8 years old), which may suggest that in some of the cases, FoH increases with greater levels of understanding of and awareness about diabetes and its management. In order to explain our finding of no significant difference in FoH between different age groups in children, the complexity of the children’s and parents’ psychology and the numerous confounding factors that may cause FoH should be considered. A high level of FoH is not necessarily always related to the individual’s levels of understanding and awareness about diabetes but more likely represents a phobia determined by multiple factors, including gender, genotype, certain personality types (e.g., avoidant, dependent, or obsessive-compulsive personality), and environmental and psychosocial factors (e.g., negative experiences and difficult relationships with family and peers) [32]. It would be reasonable to anticipate that unless these factors are eliminated or dealt with using specialist care, FoH will more likely persist long-term.

No correlation was also found between FoH and the duration of diabetes. It could be speculated that a longer duration of diabetes may suggest familiarity with diabetes management, hence, reduced diabetes-related anxiety. However, this hypothesis was not confirmed by our findings.

Furthermore, no significant differences were observed when the role of the mode of treatment in parents’ and children’s FoH was assessed, although parents of children treated via an insulin pump exhibited slightly higher HFS scores compared to those of children on MDI. Other studies have also shown that families of insulin pump-treated children did not have lower FoH, although pump therapy has been reported to be associated with lower overall diabetes-related stress in parents of pediatric patients [33]. In any case, our findings should be interpreted with caution due to the small sample of insulin pump-treated children and adolescents.

To our surprise, although a positive relationship between severe hypoglycemic episodes and FoH was anticipated, experiencing more than one severe hypoglycemic episode during the previous 12 months did not have any effect on the parents’ scores of FoH. By contrast, it did negatively influence the children’s scores for the HFS Behavior subscale and for the Total HFS scale. Conversely, previous studies have shown that the severity and the frequency of previous hypoglycemic episodes are related to the intensity of FoH [19, 23, 34, 35]. One possible explanation for this finding may be that novel advances in technology which have improved glucose monitoring may have played a role in significantly reducing the psychological burden of hypoglycemia for the parents. Real-time continuous glucose monitoring or “flash” monitoring provides information regarding glucose trends and an upcoming occurrence of hypoglycemia. Interestingly, all the studies showing a positive relationship between FoH and previous severe hypoglycemic episodes were conducted from 1992 to 2013, before modern diabetes technologies were developed and widely used. In addition, repeated severe hypoglycemic episodes may familiarize children with the condition, while previous successful management may be reassuring.

With respect to moderate episodes, more than two moderate hypoglycemic episodes during the previous 6 months did not affect parental FoH, although they were associated with a lower FoH in children. Mild hypoglycemic episodes did not have an effect on either parental or children’s FoH. It is to be noted, however, that these results should be interpreted with caution as there might be recall or report bias among the parents or the children.

Also of interest is our finding of a significant positive correlation between the Total HFS scores and the overall PedsQL™ scores, as well as the significantly higher PedsQL™ scores calculated for the upper tertiles of HFS in both children and their parents. Since higher scores in the Peds QL™ 3.0 Diabetes Module reflect worse quality of life, a positive association between increased FoH and poor quality of life is demonstrated.

The present study primarily focuses on the current impact of FoH on diabetes management. However, a crucial question is whether FoH persists long-term in children with T1DM and their parents. Literature reports are very limited regarding sustained FoH and its potential impacts. A study in adult patients with T1DM showed that in the majority of cases, FoH persisted for at least 4 years [36]; however, long-term studies in the pediatric population are lacking. In the same context, an additional aspect that warrants further exploration is the possible effect of sustained FoH on long-term physical, psychological, or mental health due to the latter’s influence on insulin dosing, physical activity, food intake, or other aspects of behavior, and on the basis that non-normative levels of FoH disrupt normal functioning and cause anxiety and tension. The widespread use of the FoH questionnaires and documentation of the FoH at different time points following systematic follow-up of the patients could shed light on the possible long-term consequences of FoH, particularly if left untreated. Continuous patient education is important so as to make patients aware of hypoglycemia-associated dangers and provide all the necessary information for appropriate management and self-control in order that severe hypoglycemic episodes and the associated fear may be eliminated before they become established. Furthermore, early identification of increased FoH may enable prompt implementation of adequate measures in order to modify behaviors that influence the development of fear. Such measures include psychotherapeutic intervention and cognitive behavior therapy, which have proven effective in adult patients with T1DM [37, 38].

In addition, the use of advanced therapeutic technologies, including continuous glucose monitoring (CGM), insulin pumps with low glucose suspend function, and automated insulin delivery may prove beneficial for the prevention, early detection, and adequate treatment of hypoglycemia as well as for the long-term well-being of patients with T1DM and their parents.

In conclusion, the present study highlights the importance of screening for FoH in children and adolescents with T1DM and their parents, confirming literature reports of an increased psychological burden for the entire family after T1DM is diagnosed. It is important to remember that patients often have limited knowledge about hypoglycemia beyond “survival skills,” which may undermine glycemic management and cause severe stress. The finding that parents reported higher levels of FoH compared to their children underlines the significance of also targeting parental fear. Increased awareness among children’s healthcare providers should be encouraged so that they focus not only on the children’s glycemic control and mental well-being but also on parental emotional stress for the purpose of offering appropriate psychological support when needed. The latter is particularly important for the implementation of a holistic therapeutic approach since this fear can result in poor diabetes management and increased psychiatric morbidity in all members of the family of a child with diabetes.

Our findings enhance the existing knowledge on the subject. However, it is important to note that associations between FoH and demographic or disease-related parameters warrant further exploration.

## Electronic supplementary material

Below is the link to the electronic supplementary material.

Supplementary Material 1

## Data Availability

The data that support the findings of this study are available from the corresponding author upon reasonable request.
