# Supplementary material for: Evaluation of the possible impact of the fear of hypoglycemia on diabetes management in children and adolescents with type 1 diabetes mellitus and their parents: a cross-sectional study
Source: Hormones (Athens). 2024 Apr 12;23(3):419–28. doi: 10.1007/s42000-024-00560-z (PMC11436422; doi:10.1007/s42000-024-00560-z)
Supplement: Supplementary file 1 — Supplementary Material 1 [file 42000_2024_560_MOESM1_ESM.docx]

| p  a  r  e  n  t  s | 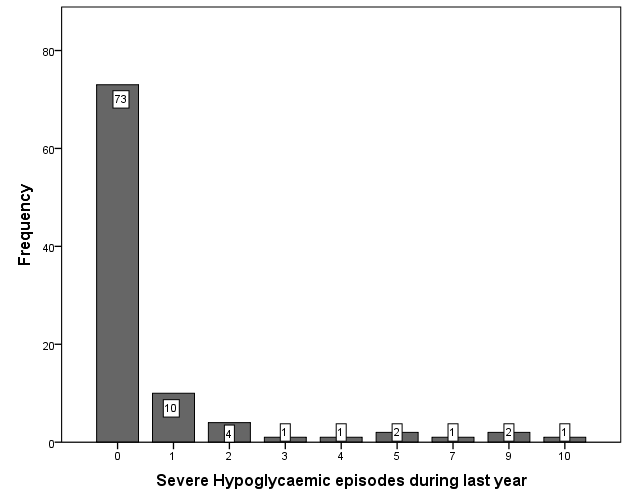 | 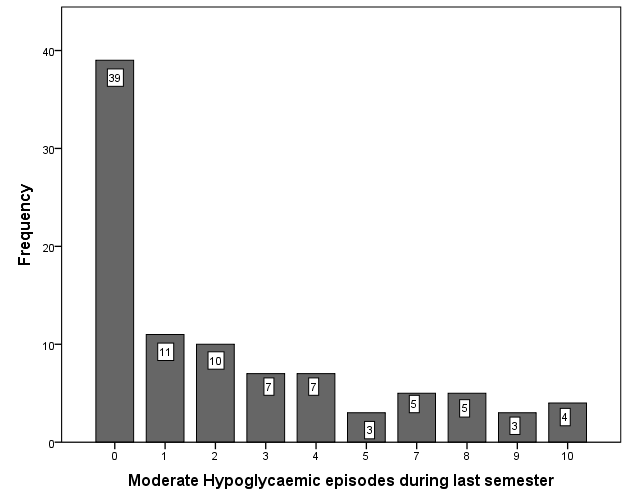 | 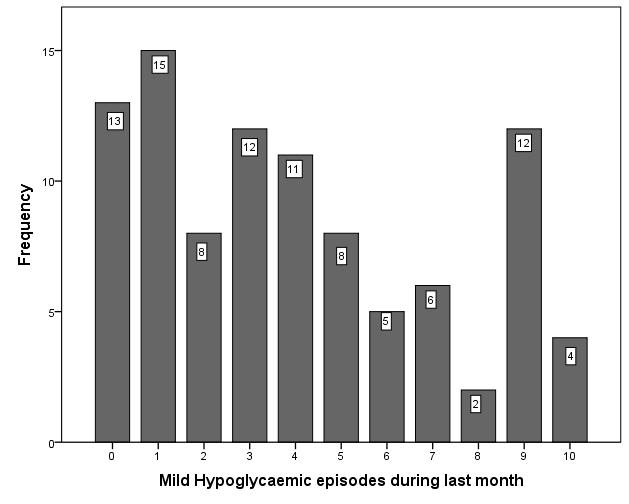 |
| --- | --- | --- | --- |
| c  h  i  ld  r  e  n | 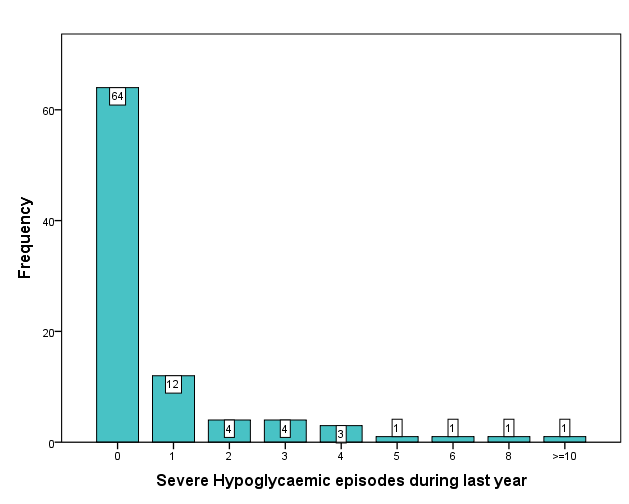 | 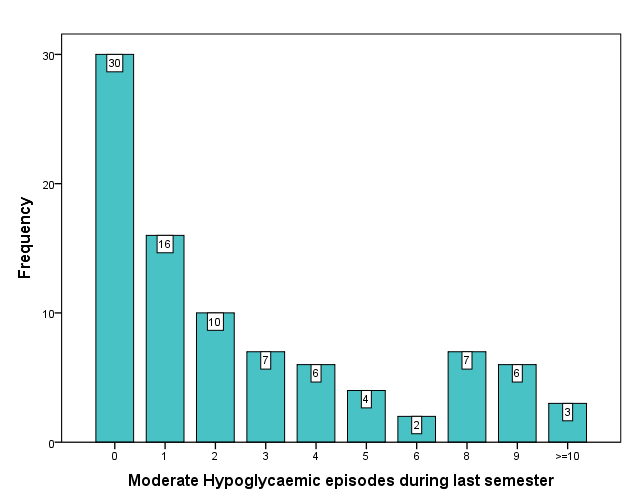 | 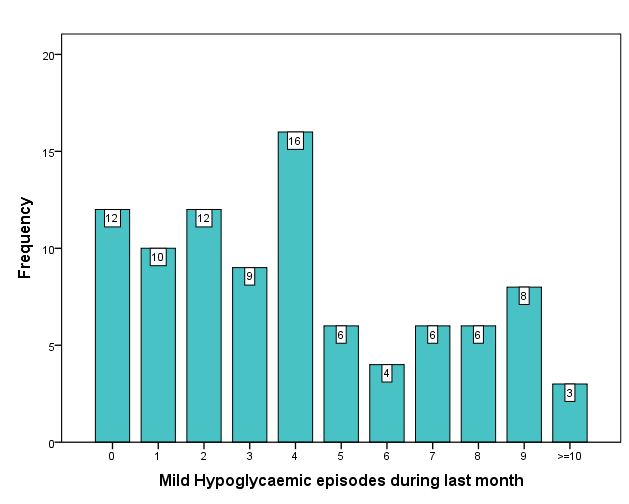 |

**Supplementary File 1** Children’s and parents’ responses regarding the frequency of severe, moderate and mild hypoglycaemic episodes during the previous year, previous 6 months and previous month, respectively.
